# Vascularized anterolateral thigh free flap for salvage reconstruction of complex anterior skull base and nasion defects after failed conventional reconstruction— how I do it

**DOI:** 10.1007/s00701-026-06895-3

**Published:** 2026-05-01

**Authors:** Hongjun Liu, Felix Knapp, Jörg Schipper, Katharina Faust, Sajjad Muhammad

**Affiliations:** 1 Department of Neurosurgery, Medical Faculty and University, Hospital Düsseldorf, Heinrich-Heine-University Düsseldorf, Düsseldorf, Germany; 2 Department of Otorhinolaryngology–Head and Neck Surgery, Düsseldorf University Hospital, Heinrich-Heine University Düsseldorf, 40225 Düsseldorf, North Rhine-Westphalia Germany

**Keywords:** Anterolateral thigh flap, Skull base reconstruction, Salvage surgery, Revision surgery, Microsurgical reconstruction, Craniofacial defect

## Abstract

**Background:**

Sinonasal inverted papilloma (SNIP) is histologically benign but locally aggressive, with high recurrence rates and a relevant risk of malignant transformation. Tumor involvement of the nasal root and overlying skin may result in a full-thickness craniofacial defect with direct intracranial–external communication. Reconstruction is particularly challenging after failure of conventional skull base techniques (e.g., titanium mesh and pericranial flaps), which may lead to pneumocephalus, cerebrospinal fluid (CSF) leakage, and infection.

**Objective:**

To describe a multidisciplinary staged salvage strategy combining bifrontal craniotomy with a vascularized anterolateral thigh (ALT) free flap for definitive reconstruction of complex anterior skull base and nasion defects after failed conventional reconstruction.

**Methods:**

We report a recurrent SNIP case with failed initial skull base reconstruction. After infection control and resolution of pneumocephalus, an ALT free flap was used. The pedicle was routed transcranially through an enlarged skull base defect to temporal recipient vessels, enabling three-layer reconstruction from dura to skin and restoration of nasal dorsum contour.

**Results:**

Definitive reconstruction was completed in 7 h. The flap survived without vascular compromise. During the postoperative course and early follow-up, no CSF leak, infection, or recurrent pneumocephalus was observed. Stable intracranial–extracranial separation and satisfactory functional/aesthetic outcomes were achieved.

**Conclusion:**

Bifrontal re-exploration followed by staged ALT free-flap reconstruction represents a viable salvage option for complex anterior skull base defects after failed primary repair. In our experience, transcranial pedicle routing and a three-layer reconstruction approach can facilitate effective intracranial-extracranial separation.

**Supplementary Information:**

The online version contains supplementary material available at 10.1007/s00701-026-06895-3.

## Introduction

Sinonasal inverted papilloma (SNIP) accounts for approximately 0.5–4% of nasal cavity neoplasms and, despite benign histology, shows locally destructive growth, recurrence, and malignant transformation potential [8]. Recurrence risk varies with tumor extent and surgical approach [4]. When SNIP involves the nasal root and overlying skin, resection may create a full-thickness craniofacial defect connecting the anterior skull base, contaminated sinonasal cavity, and external environment [5].

Failure of initial reconstruction may result in a chronically infected wound bed with CSF leakage and pneumocephalus. The ideal reconstructive solution after oncologic resection and radiotherapy must achieve several goals simultaneously: watertight separation of the intracranial and extracranial compartments to prevent CSF leak and ascending infection, obliteration of dead space, structural support of the frontal lobes, and restoration of the nasal dorsum contour and facial aesthetics [3]. For complex anterior skull base defects, free-flap reconstruction provides reliable vascularized tissue and is supported by systematic reviews [1].

Here, we describe a staged salvage technique using bifrontal craniotomy as both an approach for re-exploration and a protected corridor for microsurgical ALT free-tissue transfer to reconstruct the skull base and nasion after failed conventional reconstruction.

## Relevant surgical anatomy

The nasal root is a critical anatomical junction where the anterior cranial fossa, nasal cavity, and orbits converge. The overlying skin is thin, relatively inelastic, and often poorly suited for traditional local flap reconstruction [7]. Therefore, a vascularized flap is preferable in this setting to reduce the risk of wound-healing complications. The ALT flap, based on the descending branch of the lateral circumflex femoral artery, is well suited for this region because of its long vascular pedicle, large skin paddle, and potential for chimeric harvest including skin, fascia lata, and vastus lateralis muscle [6]. The bifrontal craniotomy provides wide exposure for resection and debridement. In this salvage concept, the anterior skull base defect created or enlarged via this approach serves as a direct channel for transcranial pedicle routing and subsequent anastomosis to superficial temporal vessels, avoiding long subcutaneous tunnels in scarred revision fields [9].

## Surgical technique and rationale for a staged salvage approach

The decision to adopt a staged salvage reconstruction was driven by prior reconstruction failure, chronic infection/contamination, and pneumocephalus. Immediate microsurgical reconstruction in such an environment carries higher risk of flap compromise and intracranial infection. A staged strategy allows infection control, resolution of pneumocephalus, and optimization of the local tissue environment before undertaking definitive reconstruction.

## Overview of the salvage strategy

The salvage strategy was implemented in two stages. The first stage focused on thorough debridement and removal of failed reconstructive materials, followed by targeted antibiotic therapy and management of pneumocephalus, with the aim of converting a contaminated wound into a stable bed suitable for reconstruction. After clinical and radiological stabilization, the second, definitive stage was performed using a vascularized ALT free flap to achieve multilayer reconstruction, including watertight dural closure, skull base support with dead-space obliteration, and restoration of the nasal root skin and contour.

## Illustrative case

**Fig. 1:**
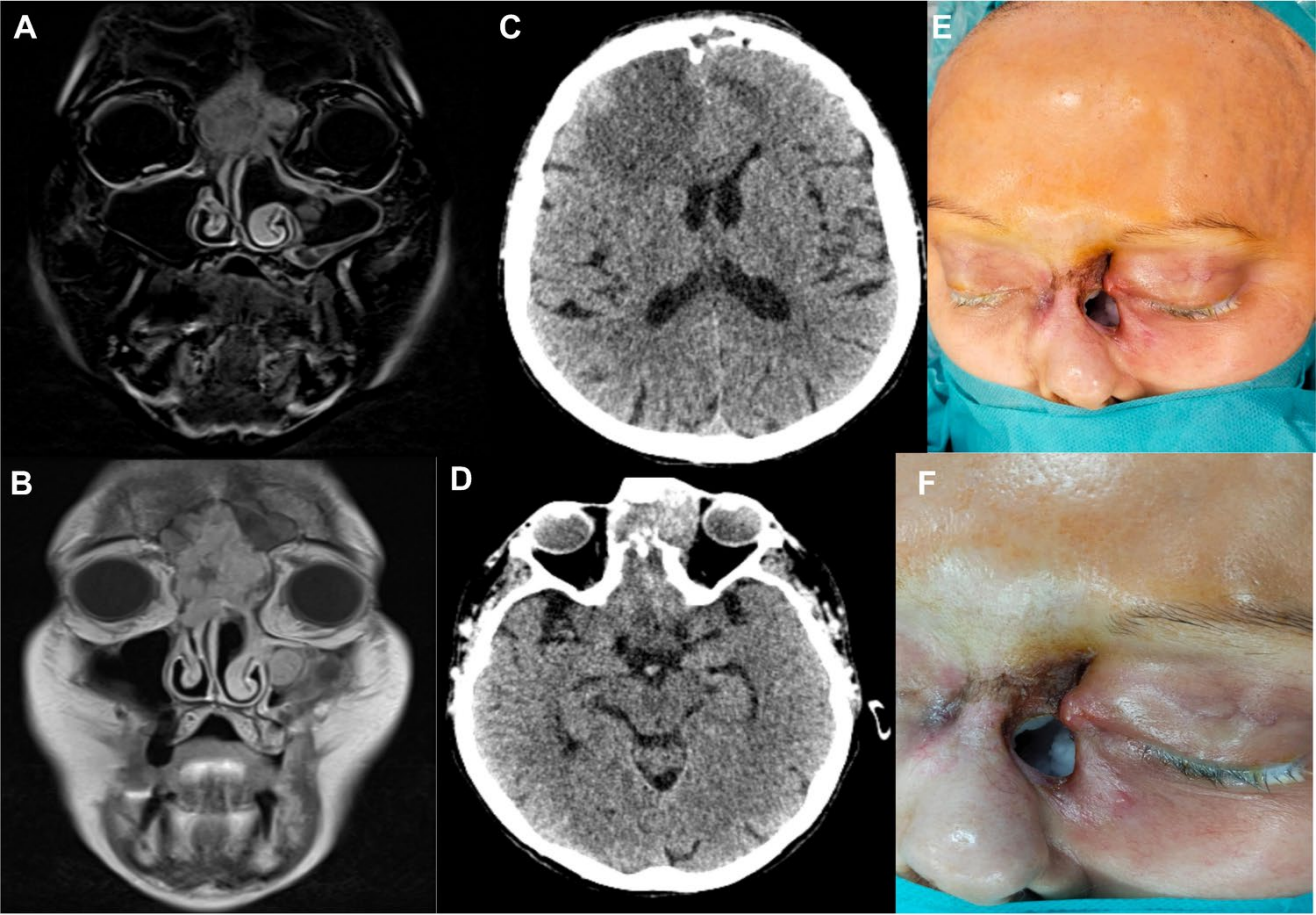
Preoperative MRI and CT of the head and clinical presentation. (**A**) Coronal MRI demonstrating a frontal mass extending inferiorly into the nasal cavity. (**B**) Axial CT showing a frontal mass with hyperdense margins. (**C**) Axial bone-window CT showing destructive changes of the nasal bones. (**D**) Sagittal bone-window CT demonstrating destruction of the cribriform plate and intracranial pneumocephalus. (**E**) Invasive tumor with nasal bone erosion and infiltration of the nasal root skin. (**F**) Direct communication between the nasal cavity and external environment after previous reconstruction failure

A 62-year-old woman was referred with recurrent SNIP and failure of a previous anterior skull base reconstruction performed at an outside hospital. The initial surgery included bifrontal craniotomy, tumor resection, reconstruction of the bony defect with titanium mesh, and coverage with a pedicled pericranial flap. This reconstruction failed, resulting in pneumocephalus, CSF leakage, and clinical deterioration (Fig. 1E, F).

Preoperative CT and MRI demonstrated extensive recurrent tumor and complications related to reconstruction failure, including destruction of the cribriform plate and planum sphenoidale, as well as intracranial air (Fig. 1A–D). The lesion exhibited aggressive growth with erosion of the nasal bones and infiltration of the nasal root skin, creating a direct communication between the nasal cavity and the external environment. Given the extensive recurrent disease and prior failed reconstruction, a salvage procedure via bifrontal craniotomy followed by the described free-flap reconstruction was planned.

## Stepwise description of the definitive salvage procedure

### Step 1: Surgical approach, recipient site preparation, and debridement

**Fig. 2:**
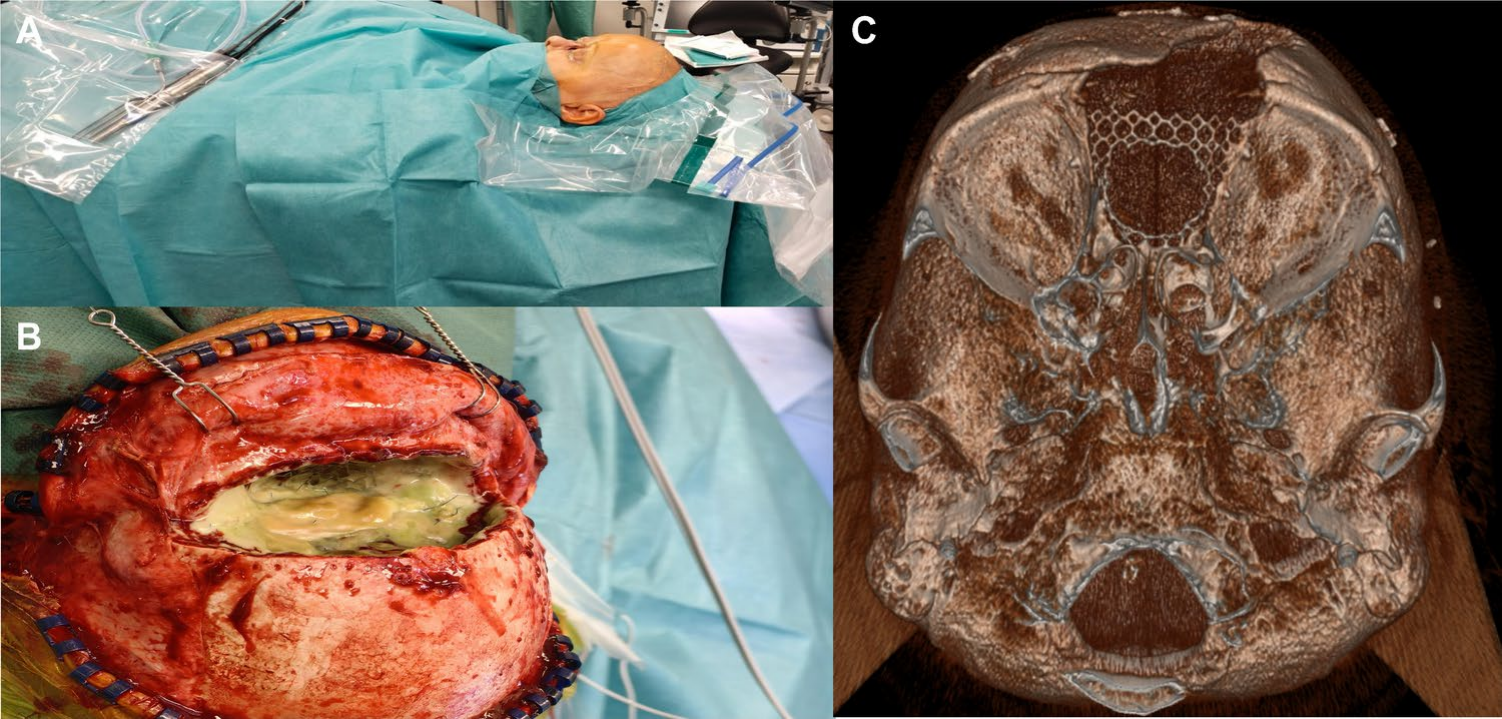
Surgical setup and key intraoperative findings. (**A**) Patient position and planned scalp incision. (**B**) Exposure of the failed titanium mesh surrounded by necrotic tissue after removal of the bone flap. (**C**) Newly placed mesh with a central window allowing passage of the reconstructive flap to the nasal root defect

The previous bicoronal incision was reopened and extended as needed (Fig. 2A). Subgaleal dissection was performed in the temporal regions, with meticulous preservation of the superficial temporal vessels and supraorbital neurovascular bundles. The frontal scalp flap was reflected anteriorly to the supraorbital rims, exposing the frontal bone and failed titanium mesh.

Existing plates and screws were removed, and the frontal bone flap elevated. Necrotic tissue and compromised titanium mesh were thoroughly debrided (Fig. 2B). A new titanium mesh, sized to restore the frontal bone defect, was prepared, and a central window was created to allow passage of the free flap skin paddle into the nasal root defect (Fig. 2C). The exposed dura was protected with TachoSil. Remaining tumor and necrotic tissue at the anterior skull base were resected where possible. Bone margins were sent for intraoperative frozen-section assessment to support achievement of an R0 resection.

### Step 2: Precise harvest of the ALT flap

**Fig. 3:**
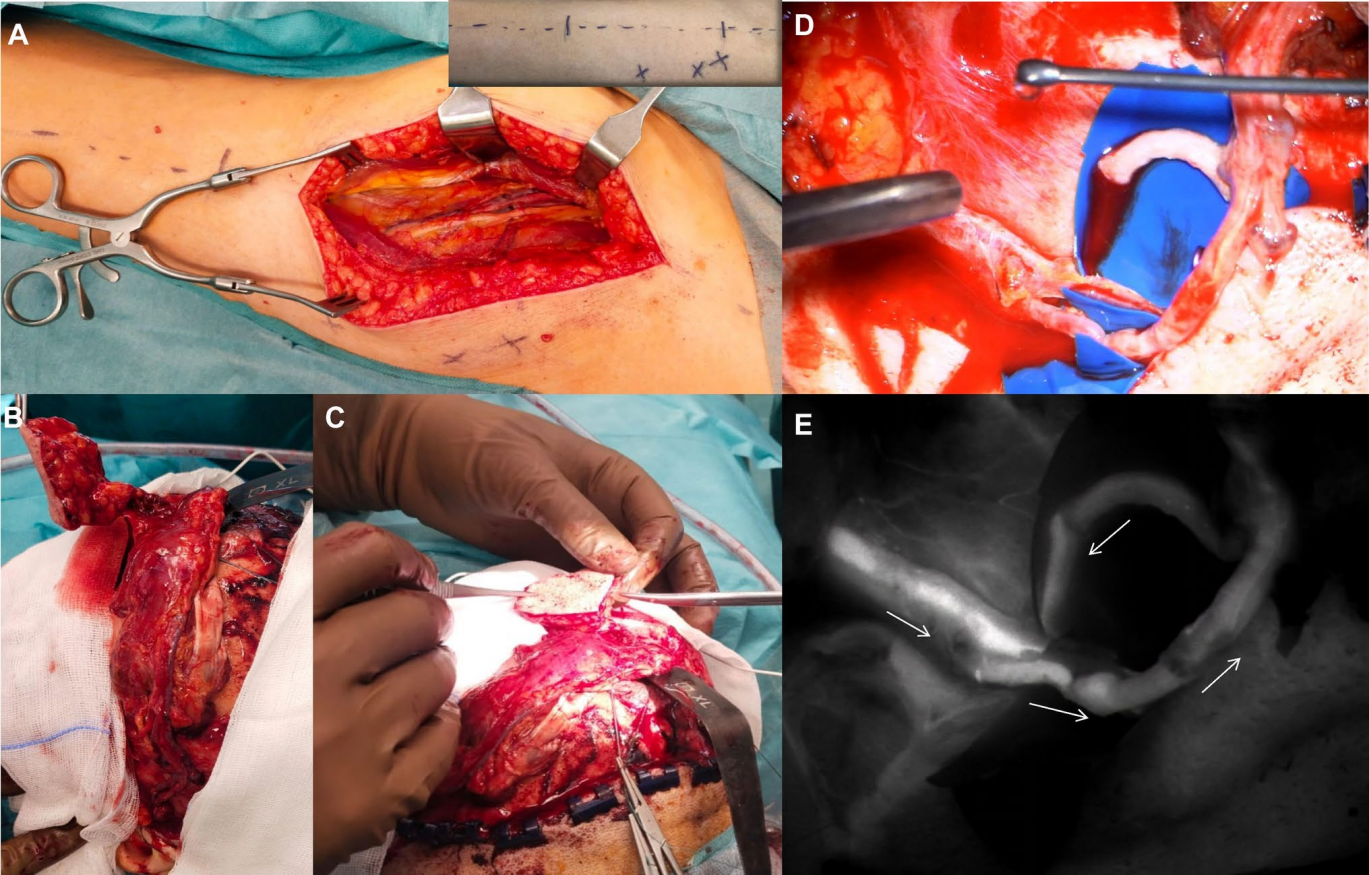
ALT perforator flap harvest and transfer. (**A**) Preoperative flap design on the lateral aspect of the left thigh based on Doppler/CTA-mapped perforators. (**B**) Intraoperative design of a perforator-based propeller flap according to actual perforator position. (**C**) Circumferential elevation of the flap, leaving an island paddle supplied by a single perforator pedicle. (**D**) Completed microvascular anastomoses (end-to-side artery, end-to-end vein). (**E**) ICG fluorescence angiography confirming good perfusion and patent anastomoses

Simultaneously, the plastic surgery team harvested an ALT flap from the left thigh. Based on preoperative Doppler/CTA mapping of perforators, a skin paddle was designed along the line from the lateral border of the patella to the anterior superior iliac spine (Fig. 3A).

Dissection proceeded through subcutaneous tissue to the fascia lata, which was incised. Under loupe magnification, the intermuscular septum and the descending branch of the lateral circumflex femoral artery with its venae comitantes were identified and traced proximally, preserving local nerve branches where possible. Depending on perforator location, a propeller-type perforator flap design was chosen, and the skin island was circumferentially elevated, leaving it attached only by the vascular pedicle (Fig. 3B, C). To reduce shear forces during transfer, the skin paddle was sutured to the underlying muscle. After completion of flap harvest, the donor artery and veins were divided and flushed with heparinized saline.

### Step 3: Recipient vessel dissection and preparation

The superficial temporal artery and venous system were exposed through an extended temporal incision. Recipient vessels were selected under microscopic visualization based on caliber match, wall quality, and flow characteristics. Venous outflow was preferentially established via the best-draining branch of the superficial temporal vein.

In revision settings or in patients with prior surgery or radiotherapy, venous drainage may be compromised due to scarring or vessel fragility. Therefore, all available superficial temporal venous branches were carefully assessed intraoperatively, and a second venous anastomosis was considered when venous return appeared borderline or insufficient. Bony edges were contoured to ensure tension- and compression-free pedicle routing along its entire course.

### Step 4: Flap inset and microsurgical anastomosis

The ALT flap was transferred to the head and neck region. The muscle portion was placed into the anterior skull base defect and ethmoidal sinus cavity, while the skin paddle was routed through the window in the titanium mesh to the nasal root defect. Particular attention was paid to ensure that the pedicle was tension-free and not twisted.

Under the operating microscope, the superficial temporal artery was clamped with Biemer clamps, and a 9–0 Prolene end-to-side arterial anastomosis was performed. The previously selected best-draining branch of the superficial temporal vein was used for the end-to-end venous anastomosis (Fig. 3D). After clamp release, the flap demonstrated strong arterial pulsation and rapid venous filling. Total ischemia time was 49 min. The pedicle was gently wrapped in temporal fascia at the bone window to protect it from friction and compression. Indocyanine green (ICG) videoangiography confirmed patency of the anastomoses and homogeneous flap perfusion (Fig. 3E).

### Step 5: Three-layer reconstruction

Dural Layer Reconstruction: The exposed dura was reinforced with TachoSil. The fascial surface of the flap was oriented toward the intracranial compartment and sutured in a watertight fashion to the dural edges with 5–0 Prolene, ensuring CSF-tight closure.

Skull Base Support and Dead Space Obliteration: The muscle component of the flap was molded to fill the ethmoidal sinus cavity and skull base defect. It was anchored to the new titanium mesh with pre-placed 2–0 Prolene sutures. Muscle volume was tailored as needed. This layered “sandwich” concept is consistent with established three-layer skull base reconstruction principles [2].

Nasal Root Skin Reconstruction: The edges of the nasal root skin defect were freshened. The skin paddle was pulled through the mesh window and inset into the nasal root defect using layered closure with 4–0 PGA and 5–0 nylon sutures, restoring contour and aesthetic subunits. Finally, the frontal bone flap was repositioned and secured.

### Postoperative management

Postoperatively, bilateral nasal packing with finger-stall gauze was maintained for 48 h, and two 12-Fr Redon drains at the ALT donor site were removed on postoperative days 4–5 depending on output. The flap was monitored daily, with the nasal root protected by a double-layer silicone foam dressing. Nose blowing was strictly prohibited, and professional nasal cavity care was provided during the early postoperative period. Continuous intravenous heparin (10,000 IU/24 h) was administered for one week, and skin sutures were removed between postoperative days 10 and 12.

Given the contaminated intracranial–sinonasal–external communication and the revision setting, perioperative broad-spectrum intravenous antibiotic prophylaxis was administered. No specific non-standard antibiotic protocol was applied. Antibiotic selection and duration followed institutional practice and were subsequently individualized based on intraoperative culture results and clinical evolution, in close collaboration with infectious disease specialists, particularly in cases with confirmed infection or removal of contaminated implants.

## Results

The definitive salvage procedure was completed as planned. The total operative time of the second stage was 7 h, including further tumor resection/debridement and reconstruction; microvascular anastomoses took approximately 40 min, and flap ischemia time was 49 min. ICG videoangiography confirmed immediate and complete flap perfusion.

**Fig. 4:**
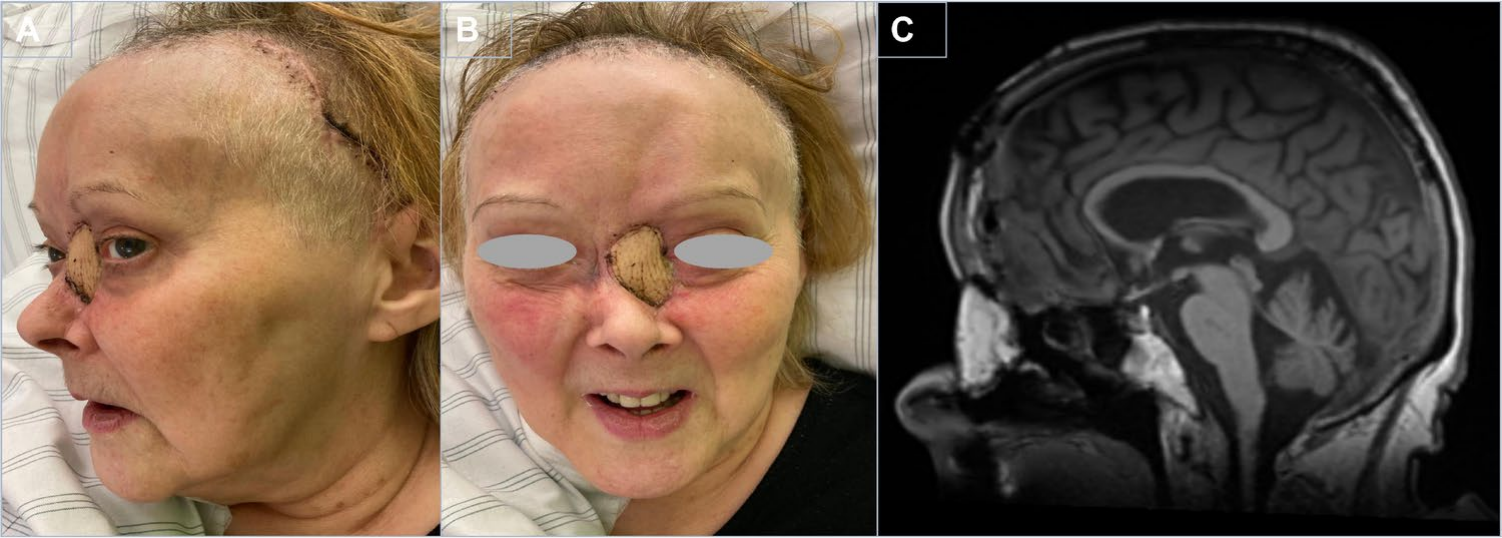
Postoperative outcome. (**A**) Well-healed reconstruction with restored contour. (**B**) Fully viable flap without vascular compromise. (**C**) MRI demonstrating typical postoperative appearance

The postoperative course was uneventful. The flap survived completely with no signs of arterial insufficiency or venous congestion. No CSF leakage, surgical site infection, or recurrent pneumocephalus occurred. At two weeks postoperatively, the reconstructed region showed stable contour and satisfactory healing with good aesthetic and functional outcome (Fig. 4A, B). An early follow-up MRI demonstrated expected postoperative changes without evidence of residual or recurrent tumor (Fig. 4C). At the 3-month clinical follow-up, the flap remained fully viable with stable integration.

## Limitations

Although the described technique offers a robust salvage option for complex craniofacial defects, several limitations must be acknowledged. First, as an illustrative "How I Do It" report based on a single case, the broader efficacy and long-term complication rates of this approach require validation in larger cohort studies. Second, the procedure is highly resource-intensive; it demands advanced microsurgical expertise and seamless multidisciplinary collaboration, which typically restricts its application to high-volume tertiary centers. Furthermore, the combined physiological burden of a bifrontal craniotomy and free-flap harvest makes this strategy unsuitable for frail patients or those with significant medical comorbidities. Postoperatively, flap monitoring presents a unique challenge: because the intracranial muscle component is buried deep within the skull base, perfusion assessment relies entirely on the visible skin paddle and adjunctive Doppler ultrasonography. Finally, patients must be preoperatively counseled regarding potential anterolateral thigh (ALT) donor-site morbidity and the frequent necessity for secondary debulking surgeries to optimize the aesthetic contour of the reconstructed nasal root.

## Strategies to avoid complications

Meticulous preoperative planning is essential, including high-resolution CTA or MRA to map ALT perforators and assess the quality of the superficial temporal vessels. Microsurgical anastomoses should be performed under microscope magnification, with interrupted sutures used to accommodate size mismatch and reduce the risk of anastomotic stenosis.

Tension- and compression-free pedicle routing must be ensured by providing adequate pedicle length and a sufficiently generous bony window, with careful inspection of the entire pedicle course. Rigorous postoperative monitoring should be implemented using standardized handheld Doppler examinations, with prompt ICG angiography and immediate re-exploration when vascular compromise is suspected.

Finally, multilayer, watertight skull base reconstruction should be achieved by exploiting the composite nature of the ALT flap, which is critical for preventing cerebrospinal fluid leakage and ascending infection.

## Patient-specific counseling

Patients should be informed this is a complex salvage procedure designed to address previous reconstruction failure and restore disease control and form/function. Counseling includes dual operative sites, operative time, recovery, donor-site scarring, sensory recovery, and possible need for secondary contouring. Long-term follow-up for oncologic surveillance and flap assessment is required.

## Key points

1. Adequate oncologic resection and infection control appear to be important prerequisites for successful salvage reconstruction.
2. In selected revision settings, a staged salvage strategy may help optimize the wound bed before definitive microsurgical reconstruction.
3. Careful preoperative planning is important to achieve adequate flap size and pedicle length.
4. The bifrontal approach can provide access for debridement and a protected corridor for subsequent microsurgical reconstruction.
5. Reliable microvascular anastomosis remains central to flap survival and may be supported by intraoperative tools such as ICG angiography.
6. The composite “sandwich” design of the ALT flap may facilitate layered reconstruction from dura to skin in selected complex anterior skull base defects.

## Supplementary Information

Below is the link to the electronic supplementary material.

Supplementary file1 (MP4 149528 KB)

## Data Availability

No datasets were generated or analysed during the current study.

## Declarations

Patient provided informed consent for use of image/video material.
